# Acceptability, feasibility and challenges of implementing an HIV prevention intervention for people living with HIV/AIDS among healthcare providers in Mozambique: Results of a qualitative study

**DOI:** 10.1080/17290376.2015.1016999

**Published:** 2015-03-17

**Authors:** Prafulta Jaiantilal, Sarah A. Gutin, Beverley Cummings, Francisco Mbofana, Carol Dawson Rose

**Affiliations:** ^a^ MSS, is a HIV Prevention Specialist affiliated to Global AIDS Program, Center for Global Health, Centers for Disease Control and Prevention, Maputo, Mozambique; ^b^ MPH, is a research scientist affiliated with the School of Nursing, Center for AIDS Prevention Studies, University of California, San Francisco, CA, USA; ^c^ MPH, is a behavioral scientist affiliated to Global AIDS Program, Center for Global Health, Centers for Disease Control and Prevention, Maputo, Mozambique; ^d^ MD, MIH is the Deputy Director affiliated to Ministry of Health, National Institute of Health, Maputo, Mozambique; ^e^ PhD, RN, FAAN is an Associate Professor affiliated to School of Nursing, Center for AIDS Prevention Studies, University of California, San Francisco, CA, USA

**Keywords:** positive prevention, feasibility and acceptability, healthcare provider, HIV/AIDS, Prévention Positive, faisabilité et acceptabilité, fournisseurs de soins médicaux

## Abstract

Despite the Mozambique government's efforts to curb human immunodeficiency virus (HIV)/acquired immune deficiency syndrome (AIDS), national prevalence is 11.5% and support is needed to expand HIV-related services and improve program quality. Positive prevention (PP) programs, which prioritize HIV prevention with people living with HIV and AIDS (PLHIV), have been recognized as an important intervention for preventing new HIV infections. To address this, an evidence-based PP training intervention was implemented with HIV healthcare providers in Mozambique. This study focuses on the acceptability and feasibility of a PP intervention in HIV clinics from the healthcare provider perspective. In-depth interviews were conducted with 31 healthcare providers from three provinces who participated in PP trainings in Mozambique. Interview data were coded using content analysis. Study data suggest that healthcare providers found PP acceptable, feasible to implement in their HIV work in clinic settings, and valued this strategy to improve HIV prevention. The PP training also led providers to feel more comfortable counseling their patients about prevention, with a more holistic approach that included HIV testing, treatment and encouraging PLHIV to live positively. While overall acceptance of the PP training was positive, several barriers to feasibility surfaced in the data. Patient-level barriers included resistance to disclosing HIV status due to fear of stigma and discrimination, difficulty negotiating for condom use, difficulty engaging men in testing and treatment, and the effects of poverty on accessing care. Providers also identified work environment barriers including high patient load, time constraints, and frequent staff turnover. Recognizing PP as an important intervention, healthcare providers should be trained to provide comprehensive prevention, care and treatment for PLHIV. Further work is needed to explore the complex social dynamics and cultural challenges such as gender inequalities, stigma and discrimination which hinder the full impact of PP interventions in this context.

## Résumé

Malgré les efforts du gouvernement mozambicain dans la lutte contre le VIH/SIDA, le taux national de séroprévalence est de 11,5% et un soutien est indispensable pour développer les services liés au VIH et améliorer la qualité des programmes. Les programmes de Prévention Positive (PP) qui mettent l'accent sur la prévention du VIH auprès des Personnes Vivant avec le VIH et le SIDA (PVVIH) ont été reconnus comme une importante mesure dans la prévention de nouvelles infections au VIH. C'est dans ce but qu'une formation de prévention basée sur les preuves a été mise en œuvre avec des fournisseurs de soins médicaux impliqués dans la lutte contre le VIH au Mozambique. Cette étude porte sur la manière dont les fournisseurs de soins médicaux perçoivent l'acceptabilité et la faisabilité d'un programme de PP dans les cliniques traitant le VIH. Des entretiens en profondeur ont été menés auprès de 31 fournisseurs de soins dans trois provinces qui ont participé à des formations à la PP au Mozambique. Les données de ces entretiens ont été codées selon la technique d'analyse de contenu. Elles suggèrent que les fournisseurs de soins médicaux trouvent la PP acceptable et applicable dans leur travail sur le VIH en milieu clinique et accordent de l'importance à cette stratégie visant à améliorer la prévention du VIH. La formation à la PP a également permis aux fournisseurs de soins médicaux de conseiller plus aisément leurs patients sur les moyens de prévention grâce à une approche plus holistique comprenant le dépistage du VIH, un traitement et encourageant les PVVIH à vivre de manière positive. Bien que la formation à la PP ait généralement été acceptée, plusieurs obstacles à sa mise en place sont apparus dans les données. Parmi les obstacles liés aux patients on trouve une résistance à divulguer la séropositivité par peur de stigmatisation et discrimination, la difficulté à négocier l'utilisation du préservatif, la difficulté d'inciter les hommes à faire des examens et suivre un traitement médical, ainsi que la question de l'accès aux soins pour les plus démunis. Ces fournisseurs de soins ont également identifié les obstacles liés à leur environnement de travail, notamment le nombre élevé de patients, les contraintes de temps et le fréquent roulement de personnel. Percevant la PP comme une mesure très importante, ces fournisseurs considèrent qu'ils devraient être formés de manière à pouvoir offrir aux PVVIH des services complets dans les domaines de la prévention, des soins et traitements. D'autres études sont à présent nécessaires pour explorer les dynamiques sociales complexes et les défis culturels tels que l'inégalité entre les sexes, la stigmatisation et la discrimination, qui dans ce contexte limitent la portée des interventions de la PP.

## 1. Introduction

Mozambique has a national human immunodeficiency virus (HIV) prevalence rate among adults 15–49 years of 11.1% (UNAIDS 2013). This translates into an estimated 1.6 million HIV-infected Mozambicans, with more than 120,000 new infections occurring annually (UNAIDS 2013). The national response to the HIV/acquired immune deficiency syndrome (AIDS) epidemic has been substantial and treatment services are now available in every district in the country. However, despite Mozambique's best efforts, only 38% of those eligible, approximately 233,000 people, are currently receiving antiretroviral therapy (ART) (National Institute of Statistics 2011). The HIV epidemic continues to increase in Mozambique, particularly in the southern part of the country where the HIV rate is as high as 25.1% (Instituto Nacional de Saúde, Instituto Nacional de Estatística & ICF Macro 2009). Continued support is needed to maintain recent achievements and expand and improve HIV-related services.

One evidence-based approach to HIV prevention is to focus prevention efforts with people living with HIV and AIDS (PLHIV) who know their serostatus. Known variously as prevention with positives, positive health, dignity, and prevention, or as commonly referred to in Mozambique, positive prevention (PP), these approaches are crucial for addressing transmission of HIV (Bunnell, Mermin & De Cock 2006; Kennedy, Medley, Sweat & O'Reilly 2010). Policy directives include PP as a cornerstone of HIV prevention efforts (CDC 2003; Kennedy *et al*. 2010). Evidence suggests that PP interventions addressing the prevention needs of PLHIV in developing countries increase the use of condoms, help keep PLHIV healthy, and prevent onward transmission while increasing PLHIV involvement in prevention efforts (Bunnell, Mermin, *et al.* 2006; World Health Organization 2007).

In the USA, PP interventions have been found to be feasible, acceptable and effective at reducing sexual behaviors that transmit HIV (Fisher, Fisher, Cornman, Amico, Bryan & Friedland 2006; Richardson, Milam, McCutchan, Stoyanoff, Bolan, Weiss, *et al*. 2004; Thrun, Cook, Bradley-Springer, Gardner, Marks, Wright, *et al*. 2009). Although the PP literature from Africa is more limited, various studies have found PP interventions to be effective at either increasing condom use or leading to a reduction in risky sexual behaviors (Bunnell, Ekwaru, Solberg, Wamai, Bikaako-Kajura, Were, *et al*. 2006; Kennedy *et al*. 2010). Fewer studies in Africa have questioned whether PP interventions are acceptable and feasible to implement. A study conducted in South Africa found that facility-based risk reduction interventions delivered by counselors for PLHIV are feasible to implement during routine clinical care and acceptable to HIV-positive patients, and may be effective at reducing unprotected sexual behavior (Cornman, Christie, Shepherd, MacDonald, Amico, Smith, *et al.* 2011; Cornman, Kiene, Christie, Fisher, Shuper, Pillay, *et al*. 2008). In addition, a cluster randomized control trial that will evaluate an HIV prevention intervention package for healthcare and treatment settings is ongoing in Kenya, Namibia, and Tanzania (Bachanas, Medley, Pals, Kidder, Antelman, Benech, *et al*. 2013; Bachanas, Moore, Bollini & Kidder 2012).

To decrease morbidity among PLHIV, prevent HIV transmission to sexual partners and children, and reduce stigma for treatment and care among patients in healthcare settings, a tailored PP intervention was implemented in Mozambique. This intervention, which is based on the HIV Intervention for Providers (HIP) approach (Dawson Rose, Courtenay-Quirk, Knight, Shade, Vittinghoff, Gomez, *et al*. 2010) was adapted through a process of key informant interviews, modifying case studies and scenarios in the training to be culturally appropriate, and then piloting of the curriculum and subsequent edits based on feedback and inputs for patients and providers. Through this process, prevention messages were tailored for the social, cultural, political and structural context of risk and HIV care in Mozambique. The curriculum was adapted to represent the realities of HIV in Mozambique including topics such as discussing disclosure, discordance counseling, family planning, prevention of mother-to-child transmission (PMTCT), and living positively.

Training materials focused on providing information and skills to healthcare providers so they could better deliver the PP intervention. A risk reduction model was used to focus on incrementally reducing transmission risks among PLHIV, with the aim of eliminating risk. A qualitative study was conducted to examine the acceptability and feasibility of integrated PP messages within routine clinical care for PLHIV from the perspective of healthcare providers who had received the PP training. This article provides an overview of the findings surrounding provider opinions about the acceptability and feasibility of PP in Mozambique.

## 2. Methods

The three-day PP training targeted healthcare providers who offer regular HIV care to PLHIV within clinical and community-based sites in Mozambique and encouraged them to address the prevention and care needs of PLHIV. The PP training program was delivered at five rural sites located in three provinces (Maputo, Sofala, and Zambézia) in Mozambique. Provinces were chosen based on high HIV prevalence rates and because they received financial support from the US President's Emergency Program for AIDS Relief (PEPFAR) for ART. With input from provincial health authorities, rural sites were selected in each province. These sites included: the Namaacha Health Center and Esperança-Beluluane Counseling and Testing Center in Maputo Province, Mafambisse Health Center in Sofala Province, and the Namacurra Health Center and Inhassunge Hospital in Zambézia Province.

The PP evaluation aimed to assess (1) the acceptability to providers of PP messages within a healthcare setting and (2) the feasibility of integrated provider-delivered PP messages in this setting. The acceptability of the PP intervention was defined as an acceptance among providers of PP as a strategy to improve HIV prevention efforts with PLHIV and discussion that the topics covered in the training were appropriate to the context of risk that providers encountered in their services for PLHIV. Feasibility was defined as the ability to integrate PP interventions and messages into regular care for PLHIV. This includes the ability to assess risk and deliver specific PP messages but also a willingness among PLHIV to engage and participate in the intervention.

Semi-structured in-depth interviews were conducted with 31 healthcare providers trained in the PP curriculum. Provider eligibility was 18 years of age or older, fluency in Portuguese, participation in a PP training workshop, and being a regular HIV care provider for PLHIV. Healthcare providers were defined as physicians, nurses, counseling and testing staff, home-based care staff, adherence support staff, support group leaders and other site staff (such as pharmacists, lab technicians and project management staff) who were trained in the PP interventions. In-depth interviews were conducted with providers to assess the acceptability of the PP training topics and the feasibility of implementing PP during routine interactions with PLHIV and also to explore barriers and facilitators to behavior change, risky or unsafe behaviors and attitudes toward PLHIV and caring for those infected. Providers were selected by the study staff using PP training attendance lists. Every other (i.e. every second) provider on the list was selected for inclusion until the minimum of five provider participants was reached at sites in Maputo and Zambézia Provinces. In Sofala Province, where trained staff came from healthcare centers, NGOs, and the government health department, providers were selected based on training attendance lists but were not all present at one clinic. Providers interested in participating in the study gave written informed consent prior to being interviewed. Providers received no monetary compensation for taking part in in-depth interviews.

Data collection took place in all three provinces from January through June 2010 and involved one round of interviews at each study site. Healthcare providers were interviewed two years after receiving the training in Maputo province, six months post-training in Sofala province and two months post-training in Zambézia province. Providers were interviewed at different times due to the expansion of the PP training program happening gradually in various regions (North, Central and South). All interviews were conducted by trained interviewers in private rooms at the sites, or in other private spaces on the site grounds. Interviewers were hired study staff members who were not affiliated with the Ministry of Health (MOH) or the PP training program. Individual interviews were conducted in Portuguese, digitally recorded and transcribed, then translated into English. Back translation was used to verify translation accuracy on a sub-sample of interviews.

Content analysis was utilized to interpret the data and focus on answering the study questions (Charmaz 2004). To ensure consistency during analysis, a codebook was developed by the study investigators to create universal definitions for each code. A team of five coders systematically worked through each transcript assigning codes throughout the text. Fifteen percent (*n* = 5) of the transcripts were double-coded to ensure inter-coder reliability of 90% or greater. ATLAS.ti (Version 6.2, Berlin, Scientific Software Development 2011), a qualitative analysis software tool, was used to manage the coding process.

Institutional Review Board approval was obtained from the Committee on Human Research at University of California, San Francisco and the Bioethics Committee for the Mozambique Ministry of Health.

## 3. Results

### 3.1. Demographics

A total of 31 healthcare providers were interviewed from the three provinces. Healthcare providers were predominantly female (*n* = 17) and 30–39 years old (*n* = 16). Table 1 presents study participants and demographics. Counselors (*n* = 19) made up the majority of healthcare providers who participated.

**Table 1.** Healthcare provider demographics (*n* = 31).

Characteristics	Total number of healthcare providers (*n* = 31)	Percentage of healthcare providers
*Gender*		
Male	14	45
Female	17	55
*Age*		
Under 30	8	26
30–39	16	52
40 and over	7	22
*Location of health center*		
Maputo Province	9	29
Sofala Province	10	32
Zambézia Province	12	39
*Occupation*		
MOH counselor/social worker	19	61
Medical technician	2	6
Nurse	3	10
Peer educators	4	13
Program manager	1	3
Pharmacist/lab technician	2	6

### 3.2. Acceptability of the PP intervention

All providers reported that addressing HIV prevention with PLHIV as well as the PP interventions and messages delivered in the training were found to be acceptable and appropriate to the context of risk that providers encountered in their services for PLHIV. The following quotes speak to this:

After this training I saw that there was really a need for this positive training, because you have to inform the HIV-positive person that they can take care of themselves at home, family members, as well as negative people, so the information I received was welcome, it enriched my share of work. (Male nurse, 43 years old, Zambézia Province)

 … as I said, in communicating with … the patient … , in addressing issues and the information that we transmit, and from the moment that … I participated in this training, I gained more experience and more motivation to continue. (Male counselor, 24 years old, Maputo Province)

They [PP messages] are very relevant to people living with HIV … to decrease the risk of new infection and reinfection … Yes, because as an infected person, you hear more about the subject, you know how it can be prevented so as to not infect someone else … . (Male counselor, 35 years old, Zambézia Province)

The PP training also led providers to feel more comfortable counseling their patients about prevention, with a more holistic approach that included partner HIV testing, treatment and encouraging PLHIV to live positively.

I benefited from it [the training] a lot, because there were many things that we were not using, we didn't talk much about Positive Prevention … we never took into account the people who were already infected, what they could do to continue to live well and protect themselves so for me it was a very useful thing, it was something that helped complete the work that I was already doing. (Female program manager, 33 years old, Sofala Province)

 … we start testing, then explain the importance of knowing their HIV status, the person accepts it, then we advise them after they take the test, the results come out, … and from there you have to accept living … with HIV and AIDS. And another thing, she has to accept to continue to use health services, to have follow-ups and receive treatment. (Female Maternal and Child Health Nurse, 43 years old, Maputo Province)

I like to advise patients to always bring their partners, to invite the partners to do the testing because with the results it is easy to prevent infection and it is easy … to avoid death. (Male Nurse, 41 years old, Zambezia Province)

In addition to the acceptance of PP as a strategy to improve HIV prevention, the PP training empowered healthcare providers to deliver prevention messages to PLHIV about reducing their risk of transmitting HIV and living positively.

### 3.3. Feasibility

The feasibility of addressing and integrating PP interventions and messages in healthcare settings that regularly serve PLHIV was also examined. Part of feasibility was the ability to discuss specific PP messages. Healthcare providers were able to implement several of the practices learned during the PP training, including risk assessment, risk reduction counseling, counseling for a reduction in the number of sexual partners, adherence to treatment, PMTCT and the importance of positive living. These elements are shown below:

I learned that while condom use is a form of prevention, treatment was also part of prevention, because there are young HIV-positive people who want to have children, but when they are not being treated it is difficult for them to have children that are not HIV-positive, so I learned that the treatment reduces the viral load and they can have children who meet all PMTCT standards, they can have HIV negative children. So it is important to encourage treatment, prevention, condom use, a change of behavior and also fidelity to one's partner. (Female medical technician, 42 years old, Maputo Province)

After training, … I had the objective of being able to counsel some patients, because there were patients who, … , were on ART, and they had not told their husbands that they were doing the ART. … I tried to advise them, and I wrote a letter requesting their spouse to appear to be able to talk to him, he could get tested, and see the result – if he was positive or not. … I could also inform the patient that they should still use condoms, because it could decrease the risk of infections in the infected patient … and the not infected patient, either way, with the positive or not positive result, they should use condoms to reduce the risk of infection. (Female counselor, 35 years old, Zambézia Province)

After the Positive Prevention training, I had good job skills, and with support from the Manual that we received … I have read and applied the knowledge gained from the Positive Prevention training. … Speaking of Positive Prevention, that is not just condom use only, but we are talking about reducing each person's risk of HIV. … With Positive Prevention we teach patients to have a good positive life and protect their wives or husbands, not wanting to distribute the virus to everyone, we explain that people should have a positive life for the sake of your health, abstinence, fidelity … I had an approach with them [HIV patients] explaining that Positive Prevention was not only condom use, but a change in the person's behavior to avoid passing the virus to others, disclose your status to sexual partners. (Male counselor, 26 years old, Zambézia Province)

Providers’ comments on how they were able to discuss the PP topics they were trained on demonstrated that integrating PP interventions into care was feasible.

Another aspect of feasibility is a willingness on the part of PLHIV to engage and participate in PP. Providers reported that following PP training, PLHIV at their sites had a favorable response to PP messages and showed a willingness to engage and participate in these PP activities, as can be seen below:

People understand the messages of prevention, I see people now who come to ask for condoms voluntarily, without [me] having to give them to them. At first people were not understanding it, but when we began to give more information about condom use, a lot of people now request condoms, and from here you can see that many people are understanding. (Male counselor, 39 years old, Zambézia Province)

The kind of progress that I see is because many now use condoms, they even come here to the clinic to ask for them, and in the counseling that I am doing, the partners are already able to bring their spouses to get tested … yes. (Female counselor, 35 years old, Zambézia Province)

 … I've noticed that some people begin to take advantage of these messages and begin to see that in the end what is important is that we cultivate and use Positive Prevention to prevent the spread of the disease. (Male Nurse, 41 years old, Zambézia Province)

There have been patients who come saying that … what you told me, I am implementing it and it is going well, it is giving me good results, after the counseling others say, I thought what you said was a joke, but now I'm getting better. This shows that the work that I am doing is going well and people are feeling the impact. (Male counselor, 39 years old, Zambézia Province)

As a result of implementing PP at their sites, providers started to see patients following up on the messages given to them. As seen in the quotes above, PLHIV were asking for condoms and bringing their spouses to be tested. Providers reported that PLHIV were taking an active role and feeling the impact of PP on their lives. This suggests that not only was PP feasible for providers to implement in their routine interactions with PLHIV, but PLHIV also showed a willingness to engage with their providers on these prevention techniques.

### 3.4. Challenges and barriers

In-depth interviews with healthcare providers revealed challenges and barriers faced in implementing PP strategies. Barriers directly affecting patients included difficulty disclosing HIV status, difficulty negotiating condom use, and low male engagement in healthcare facilities and HIV care. Providers said that patients, often women, had difficulty disclosing their status and were afraid to disclose to partners for fear of rejection, divorce, and abandonment, and to their families and communities for fear of stigma and discrimination. Disclosing HIV status in the context of a pregnancy was a particularly common concern.

The disclosure of your status is a very complicated issue which needs support … They don't disclose it to other relatives out of fear, because there are ladies … [who] come to the hospital, they take the test and know that the result is positive, so when they get home they are afraid to tell their husband … , so then she doesn't tell him. (Male counselor, 21 years old, Sofala Province)

It is very difficult for anyone to disclose their status to others, because they may experience many things including losing their partners’ love, a breach of trust and prestige in the community, discrimination and stigmatization. (Male counselor with NGO, 36 years old, Sofala Province)

While providers learned about the importance of condom use in order to decrease HIV transmission, they recognized that condom use was challenging for PLHIV. One issue mentioned repeatedly was the inability of women to negotiate for condoms within their relationships due to cultural and gender expectations:

 … she wants to [use condoms], but her husband will not accept, this happens very often with women, this is discussed very often, … ‘I want to use it but my husband will not – what can I do’ we go back to that story that the man is in charge at home … . (Female counselor, 28 years old, Zambézia Province)

 … Some say that the husband has no need to use condoms, … some because they have not revealed their status to their partner. Especially for women, they do not reveal their cases, and they have no way to require that [their partners] have to use a condom. And then, here [in Mozambique] … it is the man who decides. If the woman is HIV-positive she will never tell her husband that we will use a condom. The man has to start it and generally men do not like to come get tested. Women are always showing up to get tested. (Female counselor, 32 years old, Zambézia Province)

Providers also believe that women's fears of discrimination, abandonment and traditional gender roles constrained their ability to make safe choices. Condom use was also often linked to disclosure. If patients had not disclosed their status, providers reported that it was more difficult to ask for condom use since introducing condoms into the relationship would suggest HIV-positive status.

 … if the husband doesn't disclose his condition, he will never use condoms. Because then he will have to justify why he is using condoms. A wife who is doing treatment but hasn't disclosed to the husband will never say, let's use condoms. If I'm your husband, why condoms? (Male counselor, 32 years old, Maputo Province)

Provider's said that their patients reported that they were using condoms but routinely returned to the facility pregnant, suggesting a lack of condom use or method failure. This often frustrated providers because they wanted PLHIV to protect themselves and their partners. However, they were faced with the fact that condoms as a form of HIV prevention were less than ideal.

For example, discordant couples, you know, they come, we counsel them, maybe this man is negative and the woman is positive but they will not use condoms, … even those who are couples with the same result, many times pregnant women come … . It does not mean that a positive person cannot have them, they should have children, but … they have to see a doctor, they have to tell the doctor that they want to have kids …  (Female counselor, 28 years old, Zambézia Province)

Present in both the disclosure and condom issues from the provider's perspective is a lack of male engagement in HIV care and treatment settings. Whereas women might have received prevention messages, it was difficult to reach men with PP messages and promote change without male support.

I think it's easy to pass the message to women … who are always the first to seek my services, but [it] is more difficult to invite the male partner to actively participate. When he is not being diagnosed first … it is more difficult for the wife to get him to come. (Female maternal and child health nurse, 43 years old, Maputo Province)

What is difficult is finding men to pass on the message … If we went to see the organizations that are working in this area, … most participants are women, but at the same time we discovered that even though the woman has the information, she still does not have the power, even if the husband has the information, we need to work with men to change this. (Female program manager, 33 years old, Sofala Province)

A second type of barrier discussed by providers related to the clinic environment. These structural barriers included high patient load, time constraints, frequent staff turnover and not enough staff, and supply chain logistics issues (e.g. condom stock-outs).

The facilities are not appropriate. The time factor. Joint offices for consultations. For example, five minutes to talk to an HIV-positive patient is not enough. … we end up rushing it. (Male medical technician, 47 years old, Maputo Province)

 … In general, maybe the lack of staff [hinders Positive Prevention], … because sometimes the flow of patients into this area needs a person … I think that I needed a permanent person, … but sometimes the people [the healthcare providers] themselves are moved so abruptly, there is not enough staff for other activities. (Female Maternal and Child Health Nurse, 43 years old, Maputo Province)

 … the condoms sometimes end up running out so sometimes patients arrive at the clinic in need of condoms and they hear that there are none, and how they wanted to do something that day, they may go do it like that and do it without a condom because there are none. (Male counselor, 39 years old, Zambézia Province)

While these issues were troubling to providers, they were secondary to the many social and cultural barriers that providers described. Providers voiced many barriers that PLHIV face when trying to make safer sexual choices, but had few strategies for addressing the social and cultural challenges they described.

## 4. Discussion

In this study, PP messages and interventions were reported to be useful, relevant, acceptable and feasible for Mozambican healthcare providers. Providers also showed more comfort in counseling their patients about prevention and using a holistic approach that includes HIV partner testing, treatment and positive living. As a result of the training, providers were able to implement this new approach with their patients. This included the ability to implement or discuss several of the practices learned during the PP training, such as risk assessment, risk reduction counseling, reduction in the number of sexual partners, adherence to treatment, PMTCT and the importance of positive living.

Much like our results from Mozambique, US-based PP interventions have been feasible and acceptable to implement (Fisher *et al*. 2006; Richardson *et al*. 2004; Thrun *et al*. 2009). Thrun and colleagues in the USA have found that their Positive Steps training, a PP intervention for providers, led to positive changes in attitudes, comfort, self-efficacy, and frequency in discussing prevention issues (Thrun *et al*. 2009). Another study in the USA concluded that interventions that are well matched to the clinical environment and patient population being served were feasible and acceptable to healthcare providers, prevention interventionists and clinic staff (Koester, Maiorana, Morin, Rose, Shade & Myers 2012).

Fewer studies in Africa have questioned whether PP interventions are acceptable and feasible to implement and our work in Mozambique is among the first to look at whether PP interventions are acceptable and feasible from the standpoint of the healthcare provider. Another study looking at an HIV prevention intervention package for healthcare and treatment settings is underway in Kenya, Namibia, and Tanzania (Bachanas *et al*. 2012; Bachanas *et al.* 2013) while studies from South Africa have found that facility-based risk reduction interventions delivered by counselors for PLHIV are feasible to implement and acceptable to HIV-positive patients (Cornman *et al*. 2008; Cornman *et al*. 2011).

Despite finding that PP interventions were acceptable and feasible to providers, many providers noted social and cultural challenges to implementing PP and improved intervention uptake among patients. These challenges included patient resistance to disclosing HIV serostatus, difficulty negotiating for condoms, and difficulty engaging men. While providers recognized the benefits of disclosure, they also noted that disclosure to partners and family members was difficult for patients to implement due to fear of stigma and discrimination. Similar challenges to disclosure have also been noted in various African contexts (Greeff, Phetlhu, Makoae, Dlamini, Holzemer, Naidoo, *et al*. 2008; Medley, Garcia-Moreno, McGill & Maman 2004). Addressing stigma and decreasing inequalities between PLHIV and un-infected individuals is consistent with the Global Network of PLHIV discourse that prioritizes patient experience and concerns about stigma as a center piece in their response to addressing transmission risk behavior among PLHIV (GNP+ n.d.; GNP+ & UNAIDS 2011). It is also not surprising that fear of stigmatization affects the feasibility of prevention interventions focusing on PLHIV (Okoror, BeLue, Zungu, Adam & Airhihenbuwa 2014). It may be that the traditional disclosure support being offered is insufficient, and that psychosocial support programs in Mozambique may need to rethink the way they support couple dynamics. Couples counseling and partner testing are two alternatives that could minimize the potential negative impacts of disclosure in this environment (Medley, Baggaley, Bachanas, Cohen, Shaffer & Lo 2013). Bunnell, Meriman, *et al.* (2006) stated that disclosure, especially to partners, may facilitate effective prevention of sexual transmission of HIV, PMTCT, and treatment adherence. In Uganda, counselor-assisted disclosure for couples in their home or at a facility is being piloted, and in Kenya, partner disclosure by women with HIV has been associated with a fourfold increase in reported condom use to nearly 70% (Bunnell, Mermin, *et al.* 2006; Farquhar, Kiarie, Richardson, Kabura, John, Nduati, *et al*. 2004). Partner notification for sexually transmitted infections has been implemented in some African countries with mixed results, but could be adapted, in contextually appropriate ways, for HIV (Gaitan-Duarte, Farquhar, Horvath, Torres, Amaral, Angel, *et al*. 2014; Gichangi, Fonck, Sekande-Kigondu, Ndinya-Achola, Bwayo, Kiragu, *et al*. 2000).

Although providers understood the importance of condoms for PLHIV, they recognized that condom use was challenging. These barriers have also been cited in other African countries such as Uganda and South Africa (Allen, Mbonye, Seeley, Birungi, Wolff, Coutinho, *et al*. 2011; Cornman *et al*. 2011). The inability to negotiate for condom use within relationships, especially within established relationships, has also been noted elsewhere (Allen *et al*. 2011; Pettifor, MacPhail, Corneli, Sibeko, Kamanga, Rosenberg, *et al*. 2011). Providers felt that instructing patients to rely on condoms might not be a viable option within the context of their patients’ lives and the risk that is occurring. Allen and colleagues in Uganda came to a similar conclusion and felt that PP interventions should broaden their focus from narrow behavioral measures such as condom use and incorporate contextual and social environment factors such as economic insecurity of the family and marital status (Allen *et al*. 2011).

Issues around disclosure and condom use often involve another problem that came out in our work; difficulty engaging men in HIV prevention, care, and treatment services. In a study in Kenya, disclosure of HIV status to one's partner was identified as an important factor in determining the use of condoms and enhancing male partner involvement in making crucial decisions regarding family planning use and safe sex practices (Bii, Otieno-Nyunya, Siika & Rotich 2008). A study in Uganda among PLHIV further found that both male and female participants reported that men were the decision-makers regarding the frequency of sex and condom use and changing sexual behavior was most difficult among those who had not disclosed (Lifshay, Nakayiwa, King, Reznick, Katuntu, Batamwita, *et al*. 2009). Again, providers seem unprepared to address these complex social dynamics.

This study had limitations. The high turnover rate for healthcare providers in clinics in Mozambique, coupled with the delay between the PP training and the follow-up surveys meant that many healthcare providers who were trained were not available for follow-up interviews in order to assess their ability to implement the PP measures learned during the training. Some providers were reassigned to health centers in other provinces, others were no longer working at the clinics where they had been trained, and others were sick or on vacation. Also, the trained providers were comprised of different cadres and the sample reported on here were mostly counselors. It is possible that the results presented are not generalizable to providers who have other clinical functions in these settings.

Providers were also not all interviewed within the same timeframe after receiving training. This was due to the programmatic nature of the activities and the roll-out of training over time to various provinces. Although the providers were interviewed at different time points post-training, we did not observe any noticeable variation in the data based on time from training to evaluation follow-up; it is possible that the varying lengths of time from training to interview may have affected recall and the degree to which providers were using the PP intervention in their work. In addition, social desirability bias could have impacted the findings, causing providers to describe PP as more acceptable and feasible than it is in reality. However, to reduce this possibility, interviewers were trained study staff who were not affiliated with the PP training program or the MOH. Another challenge is that reported patient barriers, as described by providers, may be inaccurate and may not actually represent the barriers that patients face. The reported barriers listed by providers may reflect their own biases or personal challenges addressing tough issues such as sexual behavior and condom negotiation. Providers in other studies have had challenges with topics about behaviors that are stigmatized, for example, sexual activity among PLHIV and injecting drug use (Dawson Rose, Shade, Lum, Knight, Purcell & Parsons 2005). Furthermore, because PP in healthcare facilities was being implemented gradually during this time period, it is difficult to ascertain if external factors such as the lack of condoms would have impacted the feasibility of implementing PP measures.

Despite these limitations, PP interventions are an important component of HIV prevention efforts in Mozambique and are part of the national strategy (CNCS 2009). Our results demonstrate that a PP intervention approach in Mozambique is acceptable, but there are social and cultural challenges that must be addressed. Although healthcare providers face many challenges to implementing PP, they expressed the importance of reducing the onward transmission of HIV through these PP interventions. By scaling-up PP interventions, it will be possible to reach more PLHIV and this may help to slow the incidence of HIV in Mozambique.
